# Subcompartmentalisation of Proteins in the Rhoptries Correlates with Ordered Events of Erythrocyte Invasion by the Blood Stage Malaria Parasite

**DOI:** 10.1371/journal.pone.0046160

**Published:** 2012-09-25

**Authors:** Elizabeth S. Zuccala, Alexander M. Gout, Chaitali Dekiwadia, Danushka S. Marapana, Fiona Angrisano, Lynne Turnbull, David T. Riglar, Kelly L. Rogers, Cynthia B. Whitchurch, Stuart A. Ralph, Terence P. Speed, Jake Baum

**Affiliations:** 1 Infection and Immunity, Walter and Eliza Hall Institute of Medical Research, Parkville, Victoria, Australia; 2 Bioinformatics Divisions, Walter and Eliza Hall Institute of Medical Research, Parkville, Victoria, Australia; 3 Imaging Facility, Walter and Eliza Hall Institute of Medical Research, Parkville, Victoria, Australia; 4 Department of Medical Biology, University of Melbourne, Parkville, Victoria, Australia; 5 Department of Biochemistry and Molecular Biology, Bio21 Molecular Science and Biotechnology Institute, University of Melbourne, Parkville, Victoria, Australia; 6 The ithree Institute, University of Technology Sydney, Sydney, New South Wales, Australia; Bernhard Nocht Institute for Tropical Medicine, Germany

## Abstract

Host cell infection by apicomplexan parasites plays an essential role in lifecycle progression for these obligate intracellular pathogens. For most species, including the etiological agents of malaria and toxoplasmosis, infection requires active host-cell invasion dependent on formation of a tight junction – the organising interface between parasite and host cell during entry. Formation of this structure is not, however, shared across all Apicomplexa or indeed all parasite lifecycle stages. Here, using an *in silico* integrative genomic search and endogenous gene-tagging strategy, we sought to characterise proteins that function specifically during junction-dependent invasion, a class of proteins we term *invasins* to distinguish them from *adhesins* that function in species specific host-cell recognition. High-definition imaging of tagged *Plasmodium falciparum* invasins localised proteins to multiple cellular compartments of the blood stage merozoite. This includes several that localise to distinct subcompartments within the rhoptries. While originating from the same organelle, however, each has very different dynamics during invasion. Apical Sushi Protein and Rhoptry Neck protein 2 release early, following the junction, whilst a novel rhoptry protein PFF0645c releases only after invasion is complete. This supports the idea that organisation of proteins within a secretory organelle determines the order and destination of protein secretion and provides a localisation-based classification strategy for predicting invasin function during apicomplexan parasite invasion.

## Introduction

The phylum Apicomplexa includes a diverse and ancient group of obligate intracellular parasites responsible for a wide variety of major diseases of humans and animals. Among their ranks are the causative agents of malaria, from the genus *Plasmodium*, and parasites responsible for toxoplasmosis and cryptosporidiosis, significant diseases of pregnant women and the immunocompromised [1], [2]. Despite the vast range of species and tissues affected by these single-celled pathogens, they are unified by a requirement to invade host cells at various stages of their complex lifecycles and by their overall cellular architecture. Host-cell invasion is achieved by motile extracellular forms called zoites, which rely on the activity of a conserved actomyosin motor that powers cell motility [3] and the possession of a specialised set of apical secretory organelles characteristic of the phylum (reviewed in [4]).

Across the phylum several distinct mechanisms are used for invasion. Host-cell entry by haemosporidan and coccidian parasites, such as *Plasmodium* zoites (sporozoites and merozoites) or *Toxoplasma gondii* tachyzoites, is defined by the formation of a specialised tight or moving junction (reviewed in [5]). By electron microscopy (EM) this structure appears as a circular electron dense interface that forms between the parasite and host cell during entry and constitutes an aperture through which the parasite passes *en route* to intracellular infection [6], [7]. The junction functions as an intersection around which the key events for invasion are organised [8]. This includes the coordinated secretion of the apical organelles the rhoptries, micronemes and dense granules [8], [9], activation of the actomyosin motor [10] and the shedding of surface bound proteins [7], [11], [12]. The tight junction also marks the point at which a new membrane-bound cellular compartment is formed, the parasitophorous vacuole (PV), which likely arises from both host and parasite derived lipids [13], [14], [15]. This constitutes a protected cellular space within the infected host cell in which the parasite resides for the duration of its intracellular development.

However, tight junction formation is not required for host-cell traversal and is not a feature of all apicomplexan parasite host cell entry. *Cryptosporidium* spp. penetrate host cells without employing a conventional apicomplexan tight junction. Instead they stimulate host-cell actin polymerisation and formation of thin cytoplasmic extensions to invade [16], [17], [18]. While invasion still involves the concurrent formation of a PV, this compartment is nonconventional when compared to those formed by *Toxoplasma* and *Plasmodium* spp., in that while intracellular it is extracytoplasmic and separated from the host cell by an electron dense host-cell membrane [18], [19]. In contrast, the ookinete, the motile *Plasmodium* zoite responsible for colonisation of the mosquito midgut, traverses midgut epithelial cells without forming either a traditional tight junction or PV [20]. As such, tight junction dependent invasion is a distinct process intimately associated with PV formation and the absence of host-cell phagocytosis.

In spite of its distinct nature to date only a few proteins have been described that are specific to the process of tight junction dependent invasion and conserved across apicomplexan species that invade by this means. Drawing an analogy with terminology for bacterial host-cell entry [21], we refer to these here as *invasins*, to distinguish them from conserved proteins involved in either general apicomplexan cell motility [22] or adhesins involved in species or lifecycle dependent host-cell recognition [23]. Of the invasins described, several have been localised definitively to follow the tight junction constriction during entry, and therefore likely constitute the core structural basis for its formation. First identified in *T. gondii* these include the RhOptry Neck proteins, RON2, RON4, RON5 and RON8 (the latter restricted to Coccidia), which form a macromolecular complex [24], [25], [26], [27]. In support of a key role for this complex, PfRON4 has recently been shown to be important, and likely essential, for zoite invasion [28]. The micronemal protein Apical Membrane Antigen 1 (AMA1) has been shown to interact with the RON complex in both *Toxoplasma* and *Plasmodium* spp. [24], [29], [30], [31] with the combined RON-AMA1 interaction believed to span both sides of the junction. AMA1 is anchored to the zoite surface and the RON complex anchored in the host cell membrane via RON2 [32], [33], [34] with RON4 secreted inside the target host cell [8], [25]. This embedded molecular interaction between an extracellular loop of RON2 with AMA1 has been proposed as the basis for the traction potential of the junction for invasion [33], [34], [35]. However, recent demonstration of the nonessentiality of AMA1 to invasion during tachyzoite and malaria parasite liver stage invasion of host cells [28] suggests that whilst involved, this interaction may not be integral to junction structure and function.

Although several groups have confirmed conservation of the RON-AMA1 complex interaction in *Plasmodium* zoites [30], [31], [36], [37], imaging of parasites (only merozoites to date) during invasion has only convincingly localised the invasins RON4 (specifically) and AMA1 (in part) to the junction constriction during invasion [8]. Preliminary evidence suggests that RON2 may also follow this path [32], [37], [38]. Whilst parallels with *Toxoplasma* data support the importance of each of these components, and by inference the other conserved member RON5, to tight junction formation, our understanding of the broad process of tight junction dependent host cell invasion is still clearly incomplete, especially now that the essentiality of AMA1 in junction formation has come into question [28].

Several studies have used *in silico* search strategies to identify novel proteins, that are either conserved across apicomplexan genera or restricted to individual species, that function during the process of host-cell invasion or motility (e.g. [39], [40], [41]). However, to date no study has attempted to identify invasins specifically. Here we have devised just such an approach, using available genomic, proteomic and transcriptomic data to define conserved proteins that are specific to true tight junction dependent invasion but absent in motile parasite forms that either do not form a conventional junction when they invade [3] (*Cryptosporidium* spp.) or do not truly invade host cells [20] (*Plasmodium* insect stage ookinetes). Using an endogenous gene tagging strategy we have explored invasin spatial localisation in *Plasmodium falciparum* and *P. berghei* blood stage parasites, with a specific focus on high definition imaging of merozoite invasion. The strategy has identified several novel invasins, resident in multiple cellular compartments and not limited to tight junction localisation, which define distinct cellular events during the conserved process of host-cell entry.

## Results

### Integrative genomic search strategy for candidate invasins

**Figure 1:**
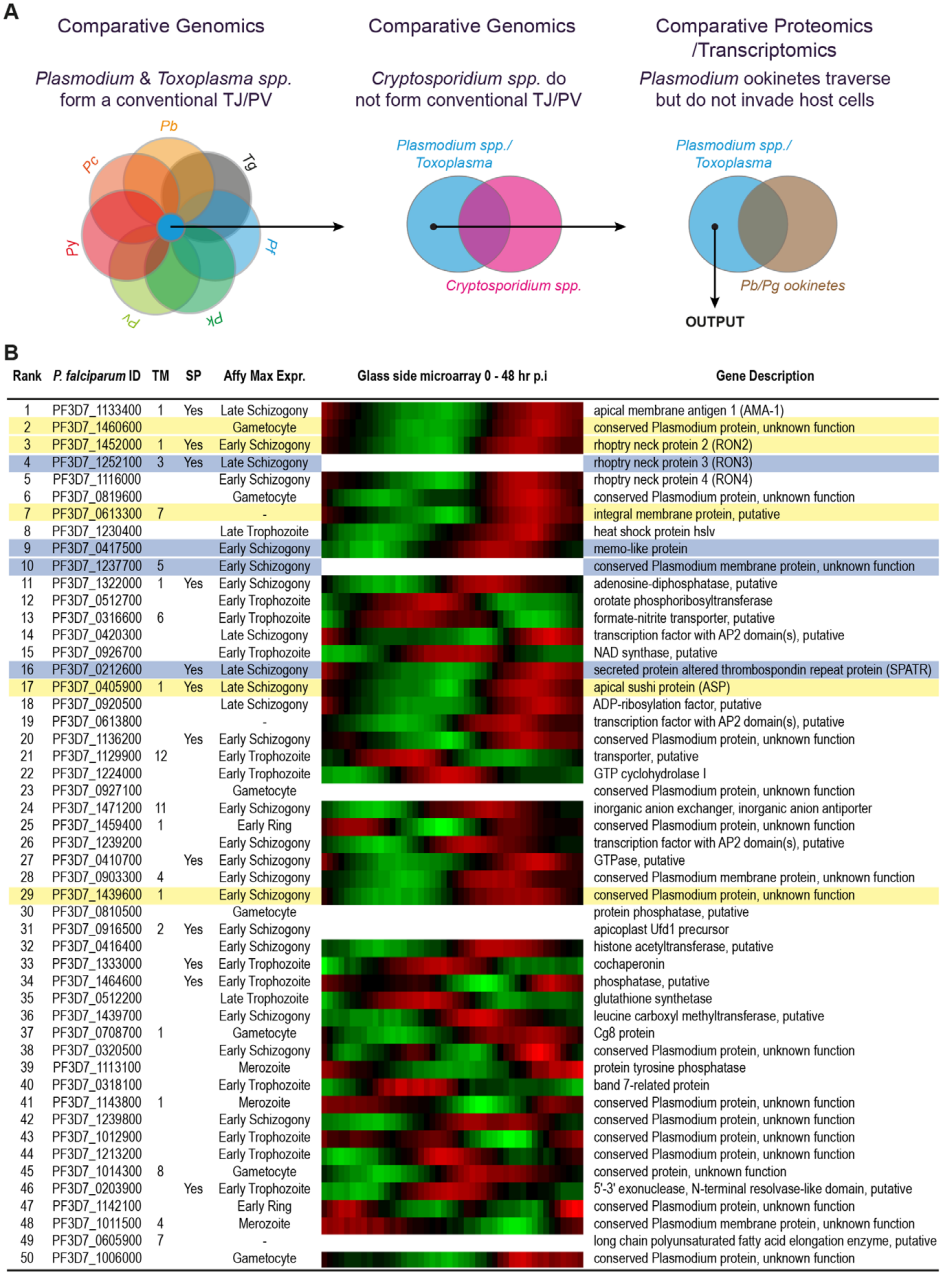
*In silico* integrative genomic search strategy to identify *P. falciparum* invasins. (**A**) To compile a list of proteins that include invasins, *P. falciparum* genes with homologues in the tight junction forming *T. gondii*, *P. berghei, P. chabaudi, P. vivax, P. yoelii* and *P. knowlesi* were selected (blue). Orthologues found in the non-tight junction forming *C. parvum* and *C. hominus* (pink) were removed from the dataset. Transcriptomic and proteomic data from *P. berghei* and *P. gallinaceum* ookinetes (brown) was used to remove proteins involved in motility but not invasion (see Table S1 and Supplemental Experimental Procedures for data sources). (**B**) The top 50 candidate invasins ranked according to *P. falciparum* asexual cycle maximum fold change in transcript expression and relative protein abundance in *P. falciparum* merozoite and sporozoite proteomes (see Table S2). Accession numbers are from PlasmoDB version 8.2. Number of transmembrane domains (TM), presence of a signal peptide (SP) and expression maximum during intra-erythrocytic cycle are listed. Heat diagram demonstrate intra-erythrocytic expression levels, given across 48 hr lifecycle with red representing high relative and green low relative expression. Proteins tagged in this study with an HA epitope are highlighted in yellow, and proteins where tagging was attempted but unsuccessful are highlighted blue.

The tight junction acts as an organising nexus where several core processes underlying apicomplexan host-cell invasion meet. However, it is not part of all apicomplexan parasite host cell entry strategies or *Plasmodium* ookinete colonisation of the mosquito midgut. To search for proteins that are specific for tight junction dependent invasion, a class of proteins we term *invasins*, we devised an *in silico* search strategy based on these general principles (Figure 1A). A primary list of proteins was assembled which have orthologues present in each of the six major *Plasmodium* species with annotated genomes (*P. falciparum, P. berghei, P. chabaudi, P. vivax, P. yoelii* and *P. knowlesi*) along with *T. gondii* but are absent in *Cryptosporidium* spp. (*C. parvum* and *C. hominus*). This resulted in a candidate list of 868 genes (including gene paralogues) out of an initial 5104 *P. falciparum* annotated genes. Genes likely involved with motility but not in the specific process of tight junction dependent invasion were then removed based on the presence of protein or transcript in *Plasmodium* ookinete proteomic and EST datasets, leaving a list of 687 candidates (Table S1). Proteins that function in the process of host-cell invasion have a gene expression profile that is often maximal in the late stages of the blood-stage asexual life cycle (during merozoite formation) and are predicted to have high relative protein abundance levels in invasive merozoite and sporozoite parasite forms. As such, genes were ranked according to each of: (i) their observed *P. falciparum* asexual cycle transcriptome maximum fold change in expression; (ii) measure of protein abundance in the *P. falciparum* sporozoite proteome and (iii) measure of protein abundance in the *P. falciparum* merozoite proteome (Table S2). A combined average of these ranks was used to arrive at a shortlist of 50 candidate genes (Figure 1B). Consideration of the asexual expression profiles within the shortlist revealed a variety of peak expression profiles. Twelve of the shortlisted 50 genes encoded a predicted signal peptide, a common feature of invasion related proteins, and 19 contained at least one predicted transmembrane domain. Of note, no candidates contained a defined PEXEL sequence, a pentameric trafficking motif common to exported proteins associated with host-cell remodeling during intra-erythrocytic development, supporting selectivity of the strategy. Furthermore, the top ten candidate invasins included known apicomplexan tight junction proteins AMA1, RON4 and RON2 as well as RON3, a protein that whilst uncharacterised with respect to invasion is implicated in tight junction dependent merozoite entry [42].

### Selection and tagging of candidate invasins in *P. falciparum*

**Figure 2:**
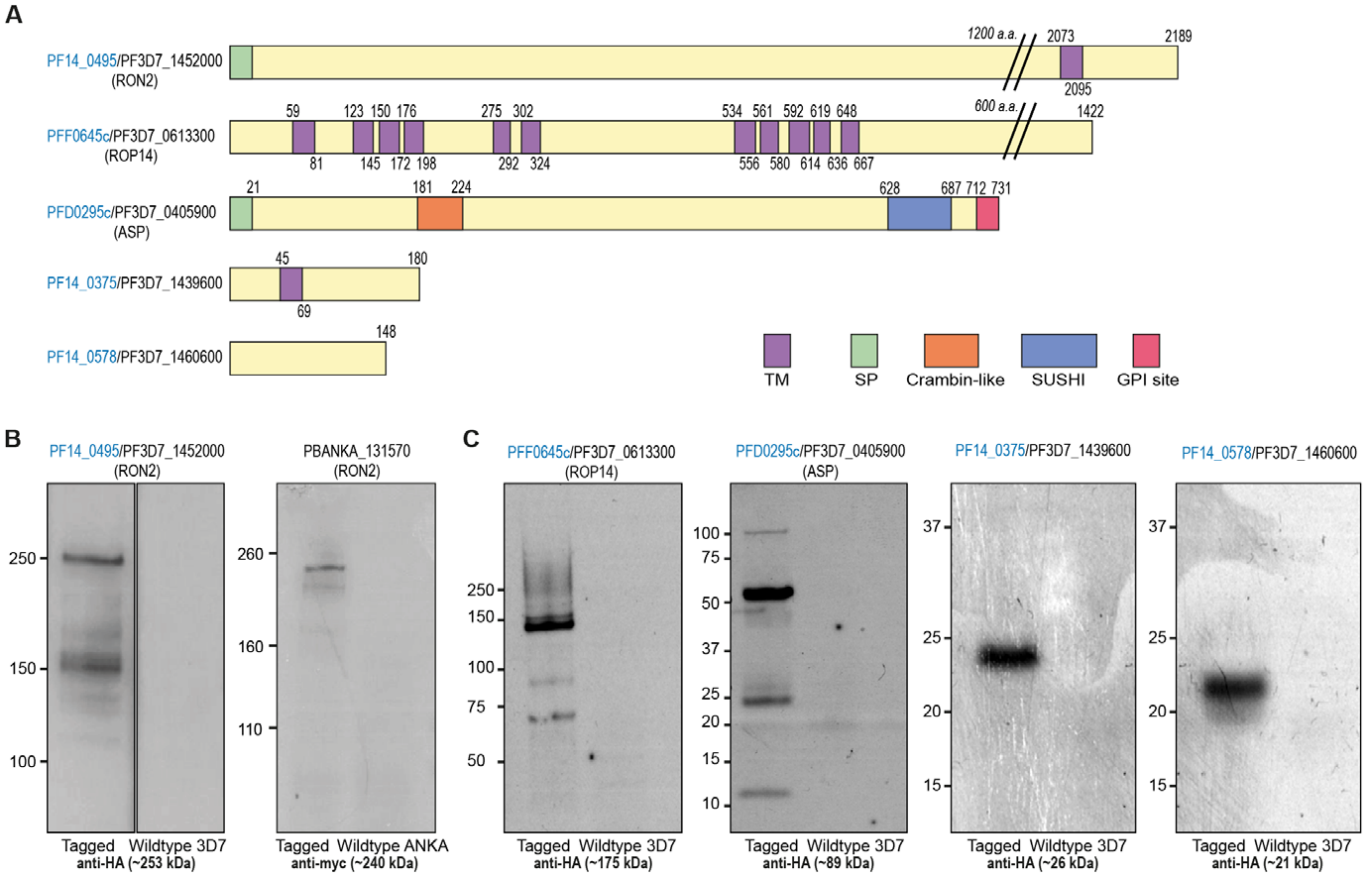
Endogenous gene tagging of candidate invasins in *P. falciparum* and *P. berghei*. (**A**) Features of the five tagged candidate invasins showing amino acid (a.a.) length, transmembrane domains (TM), signal peptides (SP), crambin-like and sushi domains and hydrophobic GPI attachment regions (GPI site). PlasmoDB version 8.2 and earlier. (**B**) Immunoblots of HA and myc-tagged parasite lines (left lanes) compared to wildtype controls (right lanes) with protein markers shown in kDa. Predicted weights of each protein (incorporating the HA tag) is given in brackets.

Upon consideration of the 50 candidate invasins, nine *P. falciparum* genes were initially selected for analysis by endogenous tagging based on their blood stage expression profiles and protein features with a particular emphasis on putative rhoptry proteins, the organelle that houses many core invasion dependent proteins (Figure 1B). This strategy produced five successfully tagged proteins for detailed cellular analysis (Figure 2A), including the already implicated junction protein PF14_0495/PF3D7_1452000, RhOptry Neck protein 2, PfRON2 (ID name is according to PlasmoDB old and new versions, see Supplemental Experimental Procedures), the hypothetical protein PFF0645c/PF3D7_0613300, a protein PFD0295c/PF3D7_0405900 previously characterised as Apical Sushi Protein (ASP, [43], [44]) and hypothetical proteins PF14_0375/PF3D7_1439600 and PF14_0578/PF3D7_1460600. Others (Figure 1B) proved refractory to PCR amplification or endogenous gene tagging, despite several attempts (data not shown).

PfRON2, originally identified in *T. gondii*, is an established component of the RON-AMA1 complex of proteins in *Plasmodium*, directly interacting with AMA1 [29], [32], [33], [34], [35], [37]. Its conserved rhoptry localisation has been confirmed in both parasite genera and evidence supports its localisation to the tight junction definitively in *T. gondii* [25], [33], [34] and tentatively during *Plasmodium* merozoite invasion of the erythrocyte [32], [37], [38]. The *T. gondii* orthologue of the multitransmembrane domain containing protein PFF0645c (TgROP14, TGME49_115220) was identified in a proteomic screen of rhoptry proteins and annotated as a possible transporter [45]. It has been suggested as a candidate for the pore that forms post zoite entry in the parasitophorous vacuole membrane (PVM) [46], however, the *Plasmodium* orthologue has remained entirely uncharacterised to date. ASP contains a sushi domain in its extracellular domain [43], a C-terminal hydrophobic GPI anchor attachment region and has been localised to the rhoptry neck [44], though its function in *P. falciparum* is unknown. The single transmembrane domain protein PF14_0375 has not been characterised in any species. PF14_0578 was recently identified as a putative *P. falciparum* inner-membrane complex (IMC) protein [39].

Integration of a 3′ triple haemagglutinin (HA) epitope into the five candidate invasin genes was confirmed by immunoblot of schizont extracts, with the size of the protein roughly consistent with expected molecular weights for all proteins, taking into account amino acid sequence and predicted overall charge (Figure 2B-C). Successful HA epitope tagging of PfRON2 at the C-terminus in *P. falciparum* mirrored a similar strategy to modify the same protein in the mouse malaria parasite *P. berghei*, where a myc epitope has previously been successfully integrated [38] both running consistent with their predicted sizes (Figure 2B). PFF0645c-HA ran as a protein product somewhat reduced in size to the expected molecular weight of 175 kDa and also showed evidence of breakdown products (Figure 2C), though their specificity could not be confirmed. Similarly, PfASP-HA appeared to run with a predominant band of a reduced size (around 50 kDa) with the presence of an additional ∼100 kDa and ∼25 kDa species. This pattern is consistent with previous findings that detected multiple PfASP fragments under reducing conditions with a dominant species at ∼50 kDa [43], [44]. Of note, C-terminal tagging of PfASP was successful despite the fact that mature protein possesses a C-terminal GPI anchor [47], a modification that would require C-terminal cleavage of the protein. This suggests that the GPI anchor may not be essential for complete PfASP function, that PfASP is dispensable for parasite viability or that successfully tagged parasites contain a pool of C-terminally cleaved and GPI modified ASP sufficient for survival but not detectable by immunoblot with anti-HA antibodies. Tagged PF14_0375 and PF14_0578 proteins ran as single products approximately consistent with their expected sizes (Figure 2C).

### Subcellular localisation of candidate invasins in intracellular parasites

**Figure 3:**
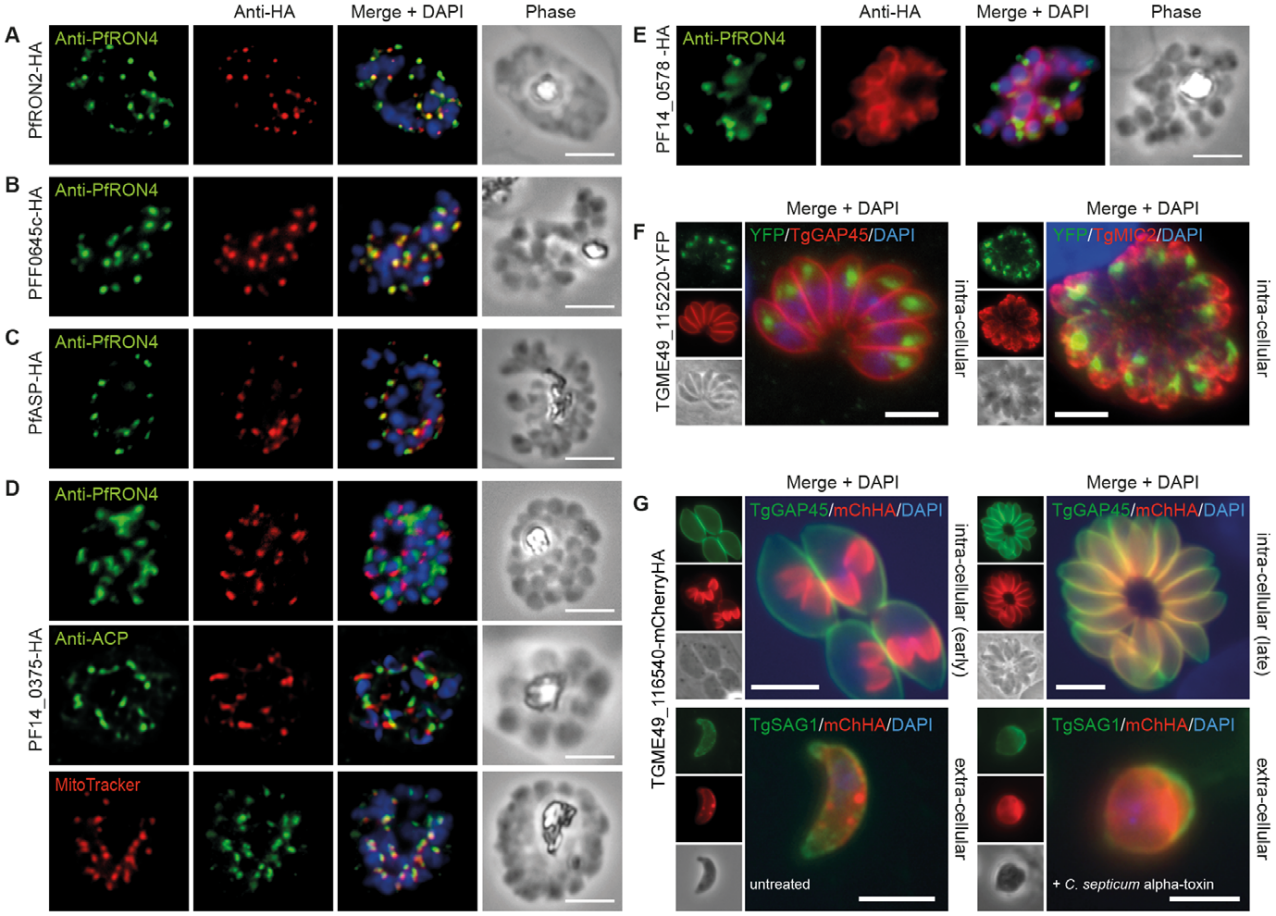
Cellular localisation of invasins in *P. falciparum* schizonts and *T. gondii* tachyzoites. (**A–E**) IFA of schizonts from HA tagged *P. falciparum* parasite lines labeled with anti-PfRON4, to mark the rhoptry neck, DAPI, to mark nuclei and anti-HA. (**A**) PfRON2 (**B**) PFF0645c (**C**) PfASP and (**E**) PF14_0578. (**D**) PF14_0375 was also colabeled with MitoTracker® Deep Red, labeling mitochondria, and anti-PfACP, labeling apicoplasts. (**F**) IFA of intracellular TGME49_115220-YFP tachyzoites (PFF0645c orthologue in *T. gondii*), colabeled with TgGAP45 (IMC) and TgMIC2 (micronemes). (**G**) Early intracellular development and extracellular TGME_116540-mCherryHA tachyzoites (PF14_0578 orthologue in *T. gondii*) colabeled with anti-TgGAP45 (IMC) and anti-TgSAG1 (surface). *C. septicum* alpha-toxin treatment swells the plasma membrane away from IMC. Scale bar = 5 µm throughout.

To determine the subcellular compartments associated with the candidate invasins, tagged proteins were localised in schizonts by immunofluorescence assay (IFA) (Figure 3A). PfRON2-HA schizonts were colabeled with anti-PfRON4, a marker of the rhoptry neck in developing merozoites [31], and demonstrated consistent coincidence of fluorescence as per previous reports [29] (Figure 3A). PFF0645c and PfASP each displayed a similar apical localisation in schizonts, showing overlap (though not complete) of labeling with PfRON4, supporting a broad rhoptry localisation (Figure 3B,C), the latter consistent with recent EM results [44]. PF14_0375-HA labeling appeared as a single point of fluorescence located on one side of developing merozoites (Figure 3D). This asymmetrical labeling (maintained throughout merozoite invasion of the erythrocyte, Figure S1) is consistent with localisation in proximity to microtubules or the closely associated organelles, the mitochondrion and apicoplast [48]. Whilst labeling with the apicoplast marker acyl carrier protein (ACP) [49] showed no overlap in labeling, mitochondrial labeling with MitoTracker® strongly implied that PF14_0375 is a mitochondrial or a mitochondria–associated protein (Figure 3D).

PF14_0578-HA labeling was peripheral in late stage schizonts (Figure 3E), suggestive of an inner-membrane complex (IMC) or plasma membrane localisation. Attempts at a definitive localisation to the IMC (with other markers) during asexual development were, however, inconclusive (data not shown).

In parallel to attempts to tag proteins in *P. falciparum*, two of the candidate hypothetical invasins were also chosen for tagging in *Toxoplasma gondii* using a recently developed endogenous gene tagging strategy [50]. We chose to focus on the uncharacterized rhoptry protein PFF0645c (TGME49_115220) and the potential IMC protein PF14_0578 (TGME49_116540) as, with the latter, the use of *T. gondii* allows definitive localisation to either the parasite plasma membrane or the IMC. The C-terminal YFP-tagged TGME49_115220 line showed a clear immunofluorescence labeling pattern at the intracellular tachyzoites apex distinct from the IMC marker GAP45 and micronemal protein MIC2 (Figure 3F), in line with rhoptry localisation [45]. In contrast, the C-terminal mCherryHA-tagged TGME49_116540 line gave a labeling pattern through intracellular growth entirely consistent with IMC development from early through late stages [51], eventually coincident with GAP45 in fully mature tachyzoites (Figure 3G, intracellular early and late). Following treatment with *Clostridium septicum* alpha-toxin, a pore-forming toxin that facilitates swelling of the plasma membrane away from the IMC [52], localisation could be clearly seen as separate and internal to the plasma membrane marker SAG1 (Figure 3G, extracellular). Thus PF14_0578 and its orthologue in *T. gondii* are IMC proteins, incorporated into the IMC during the early stages of its development before assembly of the actomyosin motor complex [52], [53].

The multiple localisations of candidate invasins (and seeming conservation across species) to different compartments in invasive apicomplexan parasites may be indicative of multiple cellular processes underlying junction dependent invasion. As a caution, the small sizes of PF14_0375 and PF14_0578 may result in their poor representation in proteomic or EST analyses, resulting in a failure to remove them from our search strategy. The role of the IMC and mitochondria in general motility would support this.

### Localisation of rhoptry proteins during merozoite invasion of the erythrocyte

To investigate the function of different invasins in erythrocyte entry, and given the large number of rhoptry proteins already implicated in host-cell invasion, we attempted high-definition imaging-based analysis on the three clear merozoite rhoptry-associated proteins PfRON2, PFF0645c and ASP. To investigate the fates of each invasin during host cell entry we localised the tagged protein using antibodies specific to the HA epitope during the process of merozoite invasion of the erythrocyte. Antibodies against the validated tight junction marker PfRON4 [8] were used to mark invading parasites and determine the stage of invasion captured.

#### Localisation of RON2

**Figure 4:**
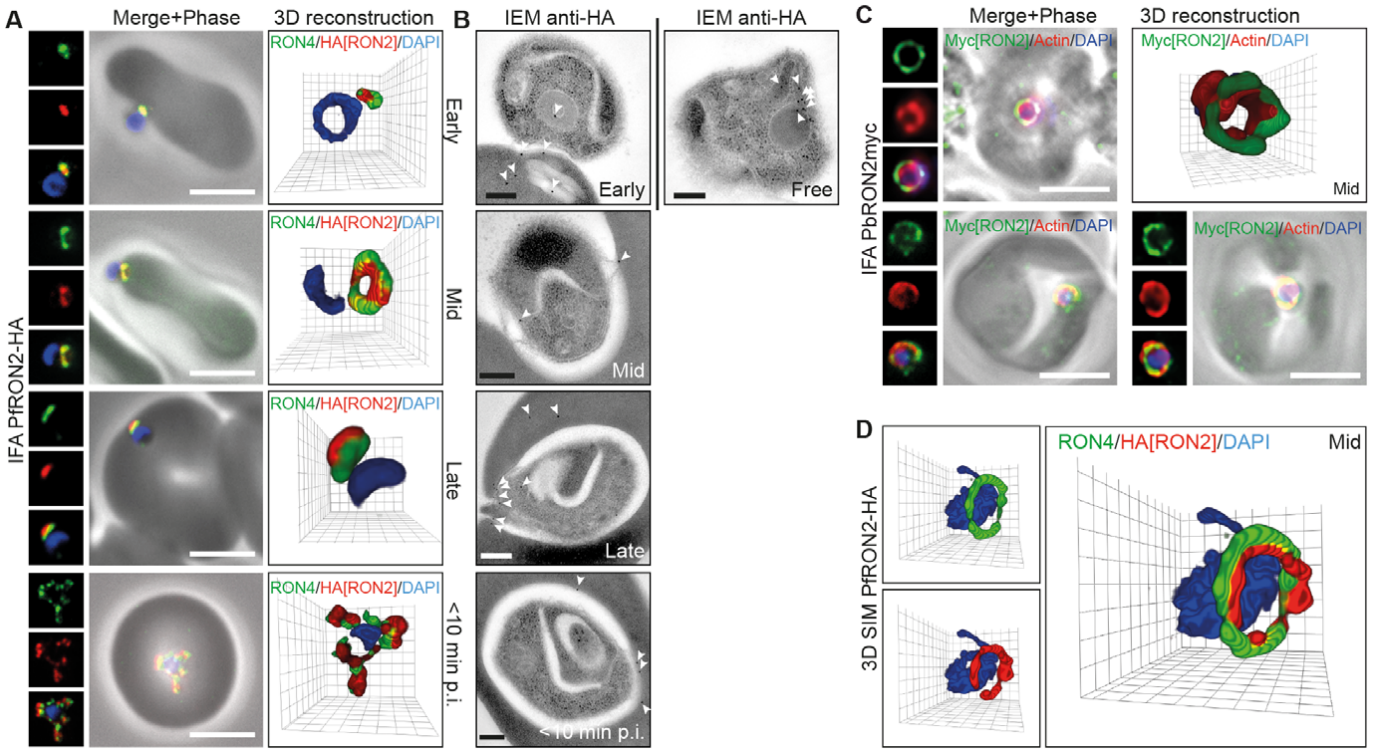
RON2 follows the tight junction during *P. falciparum* and *P. berghei* merozoite invasion. (**A**) Widefield 3D imaging of PfRON2-HA merozoites labeled with anti-HA, anti-PfRON4 (tight junction) and DAPI showing early, mid and late invasion events as well as parasites captured within 10 min post-invasion (<10 min p.i.). Scale bar = 5 µm. (**B**) IEM of free, invading and post-invasion (<10 min p.i.) PfRON2-HA merozoites labeled with anti-HA (white arrows). Scale bar = 0.2 µm. (**C**) Widefield 3D imaging of PbRON2-myc merozoites labeled with anti-myc, anti-parasite actin and DAPI captured mid invasion. IFA Scale bars = 5 µm. 3D reconstruction with 0.2 µm grid intervals. (**D**) 3D SIM of a PfRON2-HA merozoite captured mid way through invasion and labeled with anti-HA, anti-PfRON4 and DAPI. 3D reconstruction with 0.2 µm grid intervals.

In line with its invasion profile in *T. gondii*, we found that PfRON2-HA follows PfRON4 at the tight junction at all stages of invasion (Figure 4A). Early in invasion both PfRON2-HA and PfRON4 are found at the interface of the merozoite and erythrocyte at the apical tip of the parasite (Figure 4A, Early). As the merozoite moves into the forming PV, PfRON2-HA follows PfRON4 to form a circumferential zone of labeling around the parasite, which appears as a ring in three dimensions or two points of fluorescence in two-dimensional cross sections of invading parasites (Figure 4A, Mid). At the end of invasion, PfRON2-HA sits with PfRON4 at the base of the fully invaded parasite (Figure 4A, Late). Within ∼10 min post-invasion PfRON4 has been shown to redistribute to surround the new intracellular parasite where it likely sits at the erythrocytic face of the PVM [8]. PfRON2 appears to share the same fate (Figure 4A, <10 min). Each of these stages of invasion were captured by IEM using immunogold labeling of the HA epitope (Figure 4B). Of note, secretion of PfRON2-HA from the rhoptry neck could be seen with gold particles associated with either the erythrocyte plasma membrane or intraerythrocytic membranous material at the point of apical attachment (Figure 4B, Early). In one instance of invasion a pattern of labeling consistent with a ring of immunogold could be seen at the sealing tight junction (Figure 4B, Late). Following vacuolar sealing and within 10 minutes of intracellular development, immunogold labeling of PfRON2-HA was observed at the PVM. Attempts to validate the localisation by IFA using a previously published native antibody to the PfRON2 N-terminus [29], [37] were not successful under any of our fixation protocols (data not shown). However, we could extend the generality of this observation to *P. berghei* by visualising invading merozoites from a parasite line in which the PbRON2 homologue (PBANKA_131570) has been endogenously tagged with a double c-myc epitope [38]. Merozoite invasion preparations colabeled with anti-myc and an antibody that specifically recognises parasite actin (in the absence of a marker for PbRON4), which also follows the tight junction within invading *P. falciparum* and *P. berghei* merozoites [54], demonstrated a clear ring of labeling consistent with that seen in *P. falciparum* (Figure 4C).

Several groups have determined the topology of the RON protein complex interaction in *T. gondii*, where TgRON2 is placed within the host cell plasma membrane interacting with TgAMA1 at its C-terminus and presumably TgRON4 (directly or indirectly) at its N-terminus [33], [34], [35]. To explore this topology during merozoite invasion, preparations were visualised by 3D fluorescence structured illumination microscopy (SIM) [55], [56]. Mid way through invasion, PfRON2-HA clearly lies in the plane of, but within, the circumference of PfRON4 labeling when visualised by 3D SIM (Figure 4D). The localisation concentric to the PfRON4 ring is entirely in line with the topology determined in *T. gondii*, and supports the model in which RON4 is secreted inside the host cell, RON2 into the host cell plasma membrane, leaving AMA1 on the invading zoite surface [8], [25], [33], [34], [35].

Combined our data entirely validate a conserved function for RON2 as a core component of *Plasmodium*, and specifically merozoite, tight junction dependent invasion as in other Apicomplexa.

#### Localisation of PFF0645c

**Figure 5:**
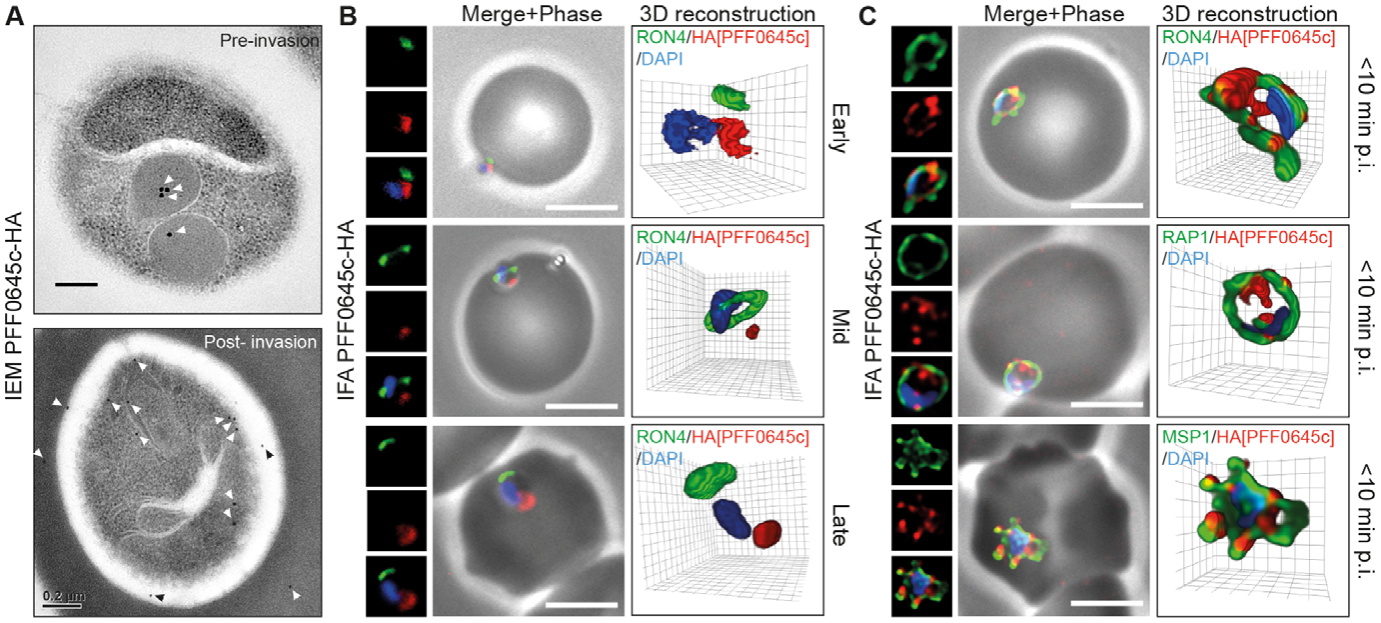
PFF0645c is released from the rhoptries only after completion of merozoite invasion. (**A**) IEM of PFF0645c-HA merozoites pre- and post-erythrocyte invasion labeled with immunogold anti-HA (white arrows). Scale bar = 0.2 µm. (**B**) Widefield 3D imaging of PFF0645c-HA merozoites labeled with anti-HA, anti-PfRON4 and DAPI showing early, mid and late invasion events. Scale bar = 5 µm. 3D reconstruction with 0.2 µm grid intervals. (**C**) Widefield 3D imaging of PFF0645c-HA early rings (<10 min post-invasion) labeled with anti-HA, anti-PfRON4 (∼PVM) anti-RAP1 (PV) or anti-MSP1 (plasma membrane) and DAPI. Scale bar = 5 µm. 3D reconstruction with 0.2 µm grid intervals.

In marked contrast to the protein redistribution seen during invasion for RON2, PFF0645c-HA originates in the rhoptry bulb (as determined by IEM, Figure 5A) and remains apical throughout invasion (Figure 5B, early, mid and late). However, post-invasion (<10 min) PFF0645c-HA was secreted to surround the intracellular parasite (Figure 5A, C). To study the location of PFF0645c-HA immediately post-invasion, *P. falciparum* merozoites were fixed within 10 min of invasion at the start of intracellular development and colabeled with PfRON4, to mark the PVM, RAP1, to mark the PV space and MSP1-19 to label the parasite plasma membrane [8]. Since PFF0645c has seven predicted transmembrane domains it is likely that its destination is either the PVM itself or the parasite plasma membrane. By IFA PFF0645c-HA surrounds the parasite, and was often observed as a series of distinct foci of labeling (Figure 5C). However, its precise membrane location could not be resolved, including via immunogold labeling where labeling to both membranes likely arose as an artifact of the fixation and dehydration process for EM (Figure 5A). Combined, this data suggests PFF0645c is the first *Plasmodium* rhoptry protein, to our knowledge, which is secreted only after zoite invasion has been completed.

#### Localisation of RON2

**Figure 6:**
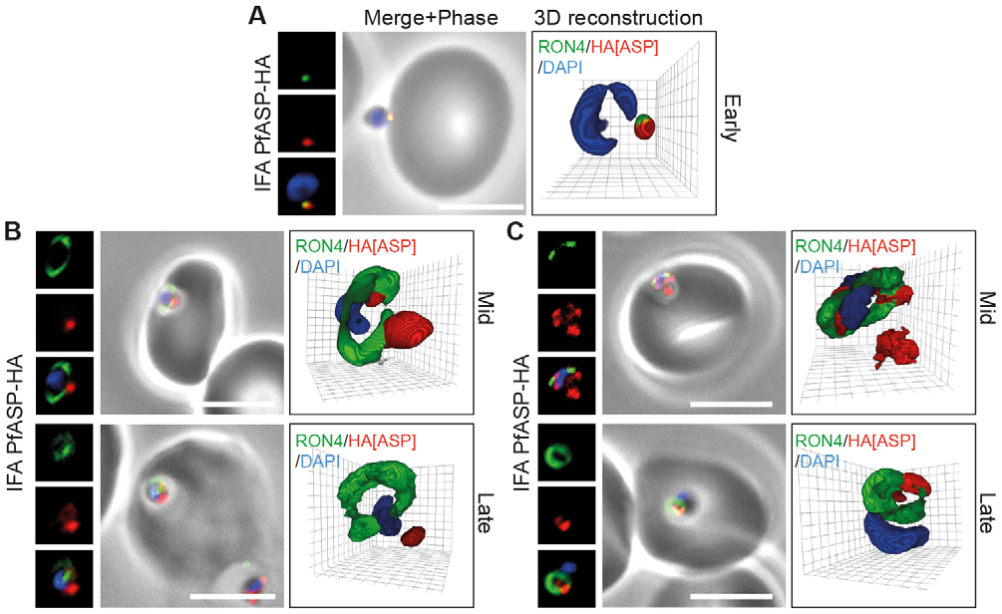
PfASP shows a dual localisation to both the tight junction and merozoite apex during erythrocyte invasion. Widefield 3D imaging of ASP-HA merozoites labeled with anti-HA, anti-PfRON4 and DAPI showing early invasion events (**A**) and two classes of mid/late invasion (**B, C**) distributions seen during invasion. Scale bar = 5 µm. 3D reconstruction with 0.2 µm grid intervals.

In apically attached parasites, PfASP-HA was located at the apical tip of the merozoite at the same point as PfRON4 (Figure 6A, Early), consistent with its localisation by IEM to the rhoptry neck in intracellular parasites [44]. Mid invasion, however, when PfRON4 labeling expands to form a ring around invading parasites at the tight junction, PfASP-HA was found either predominantly retained at the apical tip of the merozoite or associated with the tight junction (Figure 6B, C, Mid). This pattern of labeling is similar to other proteins released onto the parasite surface (including PfAMA1 and rhoptry protein Rh2 [57]), where a proportion can be left unsecreted during invasion, with the rest seen surface bound, often concentrated at the tight junction [8], [58]. Late in invasion as the tight junction moved to the base of the merozoite, PfASP-HA continued to localise either near the tight junction or at the apical tip (Figure 6B, C, Late). The absence of broad surface localisation of PfASP-HA pre-invasion, as may be expected for a GPI-anchored protein, and the inconsistency in labeling seen mid-invasion may be due to disruption of the terminal GPI anchor site at the PfASP C-terminus [47] caused by inclusion of a terminal HA epitope. These data do, however, suggest that at least a portion of PfASP is secreted from the rhoptries at an early stage of invasion and may track the junction during invasion.

### Rhoptry protein organisation reflects secretion order during invasion

**Figure 7:**
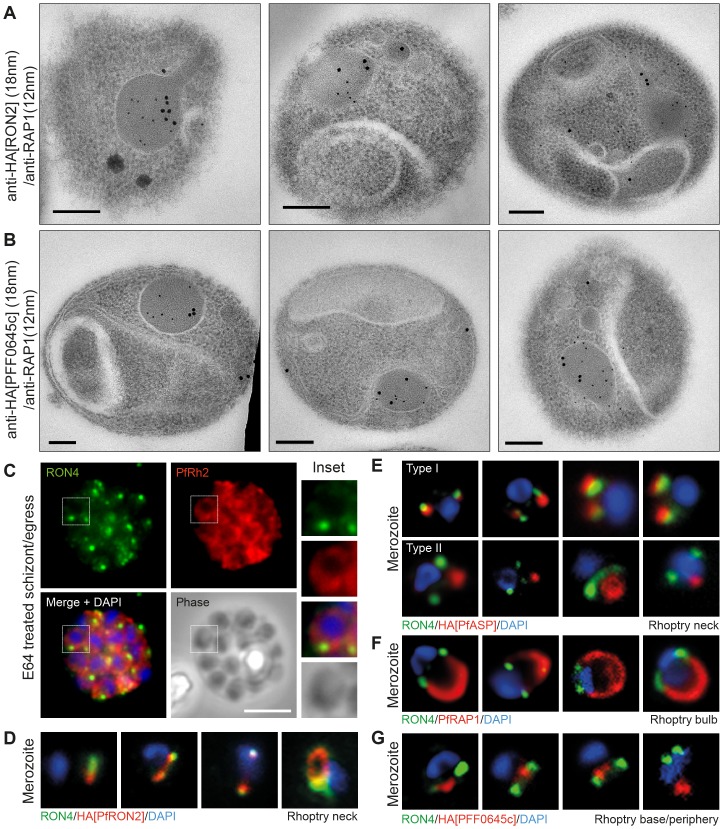
Spatial localisation of different rhoptry proteins before and during merozoite invasion. (**A**) IEM of free PfRON2-HA merozoites (pre-invasion) dual labeled with immunogold anti-HA (18 nm) and rhoptry bulb marker RAP1 (12 nm). Scale bar = 0.2 µm. (**B**) IEM of free PFF0645c-HA merozoites (pre-invasion) dual labeled with immunogold anti-HA (18 nm) and rhoptry bulb marker RAP1 (12 nm). Scale bar = 0.2 µm. (**C**) Widefield IFA of E64-treated schizonts (to prevent egress – see Materials and Methods) labeled with anti-PfRh2, anti-PfRON4 and DAPI. Scale bar = 5 µm. (**D–G**) Independent replicate imaging of merozoites from (**D**) PfRON2-HA, (**E**) PfASP-HA (two classes of distribution seen), (**F**) RAP1 and (**G**) PFF0645c-HA mid-way through invasion colabeled with anti-PfRON4 and DAPI.

**Figure 8:**
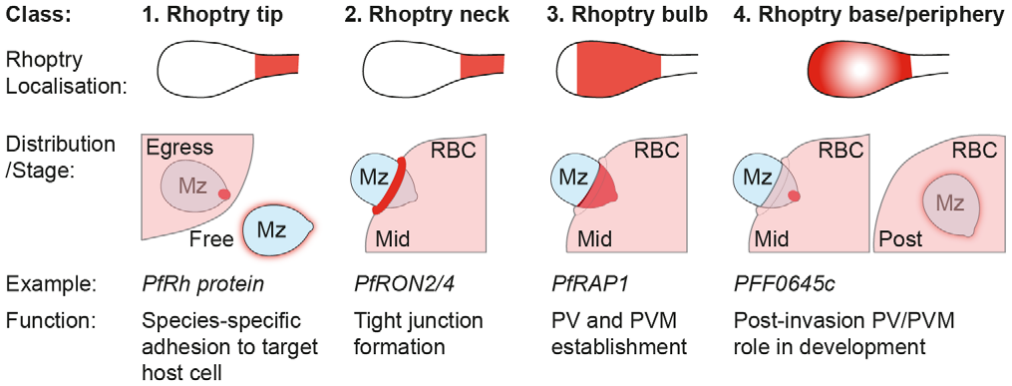
Subcompartmentalisation of rhoptries and invasin dynamics during apicomplexan host-cell invasion. A schematic for how the role that spatial distribution of rhoptry proteins pre-invasion facilitates different stages of the merozoite invasion of the erythrocyte and development post-invasion as a model for apicomplexan host-cell entry.

RON2 (from both *P. falciparum* and *P. berghei*), PFF0645c and PfASP each show distinct dynamics during merozoite invasion of erythrocytes, despite originating from the same organelle. The late secretion of PFF0645c is very different to that seen for the RON proteins and the rhoptry bulb marker RAP1 for example, each of which are secreted either early on during invasion or contiguous with PV formation [8]. This supports the idea that different localisations within the rhoptry organelle (traditionally limited to rhoptry neck and body [45]) may determine timing of release into the infected host cell. To investigate this concept further, we performed immunoelectron microscopy (IEM) on free merozoites to compare the subcompartments to which epitope tagged PfRON2 and PFF0645c reside relative to the well-characterised rhoptry body protein RAP1 [59]. Previous studies have demonstrated that PfRON2 localises to the rhoptry neck in intracellular parasites [29]. Colabeling with RAP1 in free parasites indicated that PfRON2-HA is indeed located anterior to the bulb marker, which is consistent with previous reports for PfRON4 (Figure 7A) [31], [60]. In contrast, PFF0645c had a more posterior or contiguous localisation to RAP1, frequently located towards the peripheral regions of the rhoptry (Figure 7B). Given the multiple transmembrane domains within PFF0645c, this is consistent with it either localising to the rhoptry bulb membrane or internal membrane structures at the periphery of the organelle [61]. Fluorescent imaging demonstrates that the reticulocyte binding protein homologue PfRh2, which resides at the rhoptry neck or tip [57], is secreted prior to, or at, parasite egress from the infected erythrocyte (Figure 7C). In contrast, tagged PfRON2 and PfASP, to a degree, are secreted at junction formation (Figure 4A, 6B, 7D, E), prior to release of the rhoptry bulb marker RAP1 [8] (Figure 7F). Finally, PFF0645c is only released post-invasion (Figure 5B, C, 7G). Combined, this suggests that the architecture of the rhoptry facilitates the timing of protein release and therefore the timing of establishment of key structures – the junction, nascent vacuole, and post-invasion PVM respectively (Figure 8). This supports the idea that the spatial localisation of conserved invasins, particularly those within the rhoptry, facilitates the multi-step process of parasite entry into target host cells.

## Discussion

In this study we have used both *in silico* and *in vivo* approaches for the identification and investigation of novel *P. falciparum* proteins involved in tight junction dependent host cell invasion, a class of proteins we refer to as *invasins*. Several candidate invasins were targeted for study, and five successfully tagged. Given the defined role of the rhoptry organelle in the invasion process we focused our detailed analysis on three rhoptry proteins during merozoite entry: PfRON2, PFF0645c and PfASP. Our study reveals that these proteins localise to distinct subcompartments within the rhoptry, which correlates with their timing of release and, as such, predicted function during the process of invasion.

The compartmentalisation of proteins within distinct organelle subclasses in apicomplexan zoites is an already well-characterised mechanism that facilitates the timing of key invasion dependent events. This feature is exemplified by the step-wise secretion of the core apical organelles the micronemes, rhoptries and dense granules that are broadly associated with the processes of attachment, invasion and post-invasion development respectively [8], [62]. The recent addition of the exoneme to this list, involving proteins released pre-invasion that facilitate egress, adds an additional compartmented class of proteins [63]. We now provide clear evidence that this compartmentalisation extends to within the rhoptries themselves, where spatial localisation of proteins within the rhoptry (apex/neck, body and periphery/membrane associated) is likely a key organising principle that facilitates the different roles of proteins during tight junction dependent zoite invasion.

The concept of compartmentalisation within the rhoptry is not an entirely new idea. Indeed several studies have noted the distinction between proteins that reside within the neck and body of *T. gondii* rhoptries, an observation that gave rise to the naming of ROP and RON proteins [45], and the correlation between these two compartments and the timing of secretion during invasion (reviewed in [64]). For example, the rhoptry bulb protein ROP1 is secreted to the vacuole membrane during invasion [62], [65] while the bulb proteins ROP2, ROP4, ROP5, ROP7 and ROP18 have been found at the PVM immediately post-entry, and may be secreted during or immediately following invasion [64], [66]. Although different ROP proteins lack direct orthologues among *Plasmodium* spp. and other Apicomplexa, conservation of rhoptries in all zoites that enter host cells in a tight junction dependent fashion, and their absence in the *Plasmodium* ookinete, which does not invade host cells, already points to their clear central role in the process of host-cell invasion [22]. As such, it is not surprising that an *in silico* strategy aimed at identifying invasins identifies most of the core conserved rhoptry proteins. It should be noted, however, that non-rhoptry proteins are clearly also included in this class (Figure 1), for example AMA1 [24], [31] and SPATR, both of which are micronemal proteins [67], [68].

Of the rhoptry proteins studied here, RON2 is already well established as a tight junction marker in *Toxoplasma* [25], [33], [34] and, to a limited extent, in *Plasmodium* [32], [37]. The timing of its secretion is right at the commencement of invasion concurrent with junction formation (Figure 4), a dynamic that matches the pattern for another member of the RON complex, RON4 [8]. PfASP appears to follow a similar path, variably associating with the junction during invasion (Figure 6). The localisation of both PfRON2 and PfASP to the rhoptry neck (Figure 4,6) [29], [44], and as such a position nearest to the site of release into the extracellular milieu, would fit with their early release (and therefore function) during invasion. Further work will be necessary to explore the possibility that ASP is a component of the tight junction itself. RAP1, a well characterised resident of the rhoptry bulb [8], [59], has been shown to be released only concurrent with invasion but not until the junction (or signals for its formation) has been activated [8]. Its residence posterior to the rhoptry neck again is clearly in line with the architecture of the rhoptry facilitating timing of release, with proteins further from the neck and aperture being released later. RAP1 appears to be conserved only within *Plasmodium* spp. but is likely in a similar category of proteins conserved across the phylum, perhaps analogous in function to members of the *T. gondii* ROP family of proteins [64]. PFF0645c, the orthologue of rhoptry protein TGME49_115220/TgROP14 in *Toxoplasma* [45] associates either with the membrane of the rhoptry bulb or membranous material at the periphery of the rhoptry body. It defines a relatively uncharacterised class of rhoptry proteins, clearly being released only post-invasion once the PV has been established and, given its generally posterior localisation to both RAP1 and the rhoptry neck, suggests the rhoptry bulb periphery/membrane is the last compartment to be fully secreted during invasion.

Combined with other classes of rhoptry protein (both conserved and specific to individual species) these observations establish that it is the compartmentalisation of the rhoptry into early, mid and late secreting compartments (beyond the traditional rhoptry neck and body differentiation [45]), and the organisation of proteins within these compartments pre-invasion, that facilitates the distinct stages of the stepwise process of host-cell entry.

This intuitive explanation, now established with experimental observations, is further supported by earlier data demonstrating that the merozoite invasion adhesins, the reticulocyte binding protein homolog family (PfRh), are secreted before invasion even begins [57], [58], likely prior to egress from the infected erythrocyte (Figure 7). These proteins lie within the rhoptry neck and are either contiguous with or possibly anterior to PfRON2 and PfRON4 in their spatial positioning in the organelle as determined by IEM [57], [58]. Recent data that defines a trafficking motif to the different subcompartments within the rhoptry also supports the idea that compartmentalisation is key to protein function [60]. Combining our observations here with this previous data, we propose a model in which rhoptry proteins can be divided into four broad classes based on their arrangement within the rhoptry pre-invasion and subsequent secretion profile (Figure 8): Class 1 contains those proteins such as the PfRhs that originate from the rhoptry neck and are secreted pre- or early in invasion onto the merozoite surface. Since these were not identified in our *in silico* screen, they would be expected to include those proteins associated with host-cell specific recognition and therefore distinct to each species or lifecycle stage. This fits with the functional complementarities of PfRh proteins and the micronemal adhesins the erythrocyte binding antigens (EBAs) [69]. Any protein matching their localisation would therefore be expected to be involved in host cell adhesion or recognition. The second class (Class 2) is comprised of the rhoptry neck proteins secreted following apical reorientation into the erythrocyte (data here and [8]). Since this class includes several members of the tight junction RON complex, other proteins with a similar localisation would be predicted to be either components or associated with the tight junction itself. Class 3 proteins, such as RAP1, include those proteins whose secretion from the rhoptry body into the PV is simultaneous with invasion and therefore suggestive of a role in PV establishment. The final class (Class 4) is defined by PFF0645c, which is found in the rhoptry body/membrane and remains within the merozoite until invasion is completed. Proteins that colocalise with this class of protein would be expected to function post-PV establishment, possibly functioning in either remodeling or nutrient uptake by the early-intracellular parasite. More broadly, our model presents a functional imaging-based classification strategy for further invasins based on localisation within the pre-invasion rhoptry.

The characterisation of PFF0645c and its distinct localisation and dynamics during the invasion process highlights a previously unappreciated function for the rhoptries in the establishment of early intracellular development, a function normally associated with dense granules [9]). While its precise function is unknown, the possession of at least seven predicted transmembrane domains and its homology to lipase maturation factors (via BLAST search, data not shown), a class of proteins involved in protein folding in the endoplasmic reticulum, support a role in mediating either protein trafficking or nutrient transport [46]. Further work will be necessary to define this function biochemically.

Beyond the localisation of PfASP to the rhoptries together with its apparent secretion during invasion and partial association with the tight junction, its function remains unknown. While here we found evidence for association of PfASP with the merozoite junction, it should be noted that the addition of a C-terminal tag at the endogenous locus may have disrupted GPI modification of the protein and thus impacted its localisation. PfASP also contains an N-terminal signal sequence peptide for entry into the endomembrane system, a sushi domain [43] and a crambin-like domain (PlasmoDB). Sushi domains are typically found in proteins regulating the complement system and mediate protein-protein interactions (reviewed in [70]) and crambin like domains are found in plant thionins, small toxins that attack animal cell membranes [71]. Thus, PfASP may play a role in binding host receptors during invasion along with a parallel function in creating an initial breach of, or fusion event, with the erythrocyte cell membrane to allow secretion of the RON complex into erythrocytes to initiate junction formation. This class of protein remains to be identified in *Plasmodium* blood stages in the absence of expression of a perforin-like protein in the merozoite [72].

Our demonstration that RON2 clearly traces the tight junction during both *P. falciparum* and *P. berghei* merozoite invasion confirms the conserved role of this protein across apicomplexan genera. However, while it is clear that RON2 is found at this site, its role, and indeed the role of other confirmed junction proteins, still remains unclear. For example, despite extensive functional evidence that RON2 interacts with AMA1 [33], [34], [35], the latter has recently been demonstrated as nonessential for successful *Toxoplasma* tachyzoites or *P. berghei* sporozoite tight-junction dependent invasion [28]. AMA1 still appears to be important for merozoite invasion [8], [73], where it may function predominantly in mediating early recognition and attachment events rather than in forming a core part of the junction during invasion. Nonetheless, the broad conservation of AMA1 across apicomplexan certainly supports its role in mediating conserved, rather than host-cell specific, invasion events. Notably, parasites from the apicomplexan genus *Theileria*, which do not form tight junctions and instead invade using a zipper mechanism [74], have homologues to AMA1 and RON2 [75], however the potential roles of these proteins in host cell invasion remain uninvestigated. The use of validated tagged tight junction lines, in the form of *P. falciparum* RON2-HA and *P. berghei* RON2-MYC, will no doubt prove to be invaluable tools in the elucidation of this fundamentally important aspect of invasion.

Along with the identification of proteins found in merozoite apical organelles the candidate invasin selection strategy used also implicates an IMC protein, PF14_0578, and likely mitochondrial protein or microtubule associated protein, PF14_0375, in playing conserved roles specifically in tight junction dependent invasion. Given the small size of both it is, however, possible that both constitute false positives, included because of their low abundance or below detection levels of peptide in proteomic studies with transmission stages. For example, PF14_0375 was present in the Patra *et al.* ookinete dataset [76] with a peptide count of 1, and seen therefore as a likely false-positive ookinete protein, when in fact it may have been a *bone fide* peptide linking PF14_0375 to general (but not invasion-specific) motility. If they are confirmed as true invasins the presence of an IMC protein is not wholly surprising given the known function of this structure in driving parasite motility [39], however the basis for a restricted function to invasion, and not general motility, remains unclear.

In conclusion, our study has developed an *in silico* strategy for identifying proteins that function in the restricted process of tight junction dependent invasion. Further interrogation of our list of top 50 candidates is likely to reveal other potentially key invasins. Importantly, the availability of validated tagged markers for a variety of key structures and classes of protein and their detailed description during invasion provides a strong foundation to facilitate further molecular dissection of how apicomplexan parasites, malaria parasites in particular, achieve the remarkable feat of establishing intracellular infection across such a wide variety of host species and tissues.

## Materials and Methods

### Ethics Statement

The culture of *P. falciparum* parasites using donated blood from the Australian Red Cross Society and use of mice for growing *P. berghei* have been approved by The Walter and Eliza Hall Institute Human Ethics (HEC 86/17) and Animal Ethics Committees (AEC Project 2009-023).

### *In silico* strategy to identify candidate invasins

All data sources used in the *in silico* integrative genomic, transcriptomic and proteomic search strategy are listed in Supplemental Experimental Procedures. Comparative genomic screens employed in the search strategy were achieved using phyletic queries of the OrthoMCL database (version 2) (www.orthomcl.org). Genes were selected with ortholog members present in *Plasmodium falciparum, P. berghei, P. yoelii, P. vivax, P. chabaudi, P. knowlesi* and *Toxoplasma gondii*, while absent from *Cryptosporidium parvum* and *C. hominus*. Of these, genes were removed by virtue of their presence in *Plasmodium* species' ookinete proteomic and EST datasets and mapped to *P. falciparum* genes using OrthoMCL. These datasets consisted of 1091 *P. berghei* ookinete proteins, 749 *P. gallinaceum* ookinete proteins and 345 *P. berghei* ookinete microneme proteins. Microneme proteins classed as ‘probable contaminants’, ‘probable blood stage contaminants’ or ‘blood stage contaminant confirmed by IFA’ were not considered [77]. Proteins involving a single peptide in *P. gallinaceum* ookinete datasets were not considered, given the reported false positive rate of 38.9% (Patra *et al*., 2008). *P. berghei* ESTs were sourced from an *in vitro* transformed *P. berghei* ookinete EST library and were mapped to *P. berghei* transcripts (PlasmoDB version 5.5) using BLAST prior to identifying *P. falciparum* orthologs using OrthoMCL (Table S1). Following removal of ookinete genes, candidate invasins were ranked according to their maximum fold change (MFC) of transcript abundance during the asexual blood stage. This rank comprised the average of separate ranks derived from the MFC observed during the Affymetrix and two-colour microarray time-course studies. Candidate invasins were also ranked according to their exhibited protein abundance (peptide coverage) within the *P. falciparum* merozoite proteome (Leiden Malaria Group, *unpublished*) and protein abundance (emPAI score) within the *P. falciparum* sporozoite proteome. An average of these three rankings (MFC transcript abundance, merozoite protein abundance and sporozoite protein abundance) was used to select the top 50 candidates (Table S2), which were manually curated for the presence of a N-terminal signal peptide using SignalP, transmembrane domains using TMHMM and the possession of a pentameric PEXEL motif using ExportPred. Reported subcellular localisations were retrieved from ApiLoc.

### Parasite culture and maintenance

*P. falciparum*, *P. berghei* and *T. gondii* parasites were each maintained using standard culturing procedures. *P. falciparum* cultures were grown in human O+ erythrocytes at 4% hematocrit with 0.5% wt/vol Albumax II (Life Technologies). Cultures were maintained in synchrony using 5% Sorbitol treatment or via treatment with 30 IU (approximately 230 µg/mL) heparin (Pfizer) [12]. All tagged *P. falciparum* parasites were derived from 3D7, a clone itself derived from NF54, provided by the late David Walliker at Edinburgh University, UK. For imaging with RAP1 (see below) a line derived from *P. falciparum* D10 were used [12]. *P. berghei* parasites were maintained in Balb/c mice using standard protocols. The PbRON2-myc line used [38] was generously provided by Robert Sinden, Imperial College London, UK. *T. gondii* parasites were propagated in human foreskin fibroblasts (HFF, ATCC biological resource centre) grown in Dulbecco's modified Eagle's medium (DMEM) with 1% fetal calf serum (GIBCOBRL). For α-toxin treatment, needle passaged tachyzoites were incubated in 50 µL of *Clostridium septicum* culture supernatant (generously provided by Dena Lyras and Anjana Chakravorty Monash University, Victoria, Australia) and prepared as described [52]. Tagged *T. gondii* parasites were derived from the ΔKu80 line [50].

### Zoite invasion preparation

Blood stage *P. falciparum* and *P. berghei* (ANKA) parasites were cultured through to schizogony and prepared for merozoite invasion by filtration through a 1.2 µm, 32 mm syringe filter (Sartorius Stedim Biotech) as described [8], [12] and incubated with human or mouse erythrocytes prior to fixation. To prevent complete schizont rupture, *P. falciparum* schizonts were treated with 10 µM of the cysteine protease inhibitor E64 (Sigma) for 6–8 hr.

### Cloning of tagging constructs and transfection

For endogenous tagging of *P. falciparum* invasin candidates with a 3′ triple haemagglutinin (HA) epitope, flank sequences encoding 450 bp–1100 bp of candidate invasin genes were amplified by PCR from 3D7 gDNA using specific primers (available on request) and cloned into the *BglII*-*PstI* cloning site of the pD3HA vector. This vector is derived from the pARL vector, which carries the *hDHFR* resistance cassette for drug selection [78], where a triple haemagglutinin (3*HA) tag is placed upstream of the (*XhoI* site) 3′ untranslated region of *PbDHFR*. Cloning PCR products with an addition 3′ guanine residue (XXg) into the *BglII*-*PstI* site results in a read through at the C-terminus of the invasin of interest to encode a triple alanine spacer followed by the triple HA tag: *agatct*XXg*ctgcag*caTACCCGTACGACGTCCCGGACTACGCTGGCTATCCCTATGATGTGCCCGATTATGCGTATCCGTACGATGTTCCAGATTATGCCGCTtaaccatggtgacgtcaccggtt*ctcgag* (AAAYPYDVPDYAGYPYDVPDYAYPYDVPDYAA). *P. falciparum* transfection followed standard protocols [78]. *T. gondii* proteins were tagged at the 3′ end of the endogenous gene using the ΔKu80 parasite line [50] where a 3′ flank of the candidate gene was PCR amplified from *T. gondii* RH strain genomic DNA (primers available on request) and inserted into pmCherryHA3.LIC.HX (generously provided by Christopher Tonkin, Walter & Eliza Hall Institute) or pYFP.LIC.HX vectors by ligation independent cloning strategy [50]. Plasmids were linearised within the gene flank for efficient homologous integration after transfection with integrants selected and maintained according to standard protocols.

### Western Blotting

Saponin lysed parasite pellets from late schizont stage *P. falciparum* and *P. berghei* cultures were separated in sample buffer by SDS-PAGE using either 4-12% bis-tris or 3–8% tris-acetate NuPAGE® gels (Life Technologies) under reducing conditions and transferred to nitrocellulose membranes using an iBlot® (Life Technologies) or wet transfer protocol (Whatman). Membranes were blocked in 0.1% Tween 20-phosphate-buffered saline (PBS) with 10% (w/v) skim milk and probed with primary antibodies rat anti-HA clone 3F10 (Roche) [1∶500] or mouse anti-c-Myc clone 9E10 (kindly provided by Kaye Wycherley, WEHI Monoclonal Facility, Melbourne, Australia) [1∶2000] and horseradish peroxidase (HRP) conjugated secondary antibodies rabbit anti-rat HRP (Dako) [1∶1000] or goat anti-mouse HRP (Millipore) [1∶1000] diluted in 0.1% Tween 20- PBS with 1% (w/v) skim milk. Immunoblots were developed using enhanced chemiluminescence (ECL, Amersham).

### Immunofluorescence assay (IFA), three dimensional structured illumination microscopy (3D SIM) and immuno-electron microscopy (IEM)

*Plasmodium* samples were processed for IFA in solution (invasion assay samples) or on slides (schizont cultures) as described [79]. Briefly, samples were fixed in 4% paraformaldehyde 0.0075% gluteraldehyde (Electron Microscopy Sciences) in PBS for 30 min, incubated with 0.1% (w/v) Triton-X-100 (Bio-Rad) in PBS for 10 min, blocked in 3% (w/v) bovine serum albumin (BSA, Sigma) in PBS overnight at 4°C (invasion assay samples) or for 1 hr at room temperature (schizont samples) and incubated with primary and secondary antibodies diluted in blocking solution for 1 hr (Supplental Experimental Procedures). Samples were settled onto type 1.5 coverslips (Zeiss) coated with 1% w/v polyethyleneimine (PEI) in water (invasion assay samples) and mounted in 0.1 ng/µL 4′,6-Diamidino-2-phenylindole dihydrochloride (DAPI) in VectaShield® (Vector Laboratories). For mitochondrial imaging, schizonts were purified over a magnetic column (Miltenyi Biotec) and incubated at 37°C for 30 min with 100 nM MitoTracker® Deep Red (Life Technologies) in complete culture media. Cells were washed once in PBS, pelletted, smeared onto slides and processed for IFA. Free *T. gondii* tachyzoites from culture supernatant or infected host cells were fixed on coverslips in 4% paraformaldehyde (Electron Microscopy Sciences) for 10 min, permeabilised in 0.1% Triton X-100/PBS for 10 min, and blocked in 3% BSA/PBS for 1 hr. Incubation with primary antisera in 3% BSA/PBS was for 1 hr followed by four 5 min washes in PBS and subsequent incubation with secondary antibodies (Supplemental Experimental Procedures). Fluorescence microscopy was undertaken using an inverted Axiovert 200 M motorized microscope (Zeiss) via a Plan-Apochromat 100×/1.40 NA Ph3 oil immersion lens (Zeiss) and equipped with an AxioCam MRm camera (1388×1040 pixels, Zeiss). Illumination was from a mercury lamp and light was collected via high efficiency filters from Zeiss, Ex 365/Em 445/50 (DAPI), Ex 470/40/Em 525/50 (Alexa488) and Ex 587/25/Em 647/70 (Alexa594). Where appropriate, Z-stacks were taken well above and below parasites and images were corrected for effects of chromatic aberration and underwent deconvolution (fast iterative or iterative setting) using Axiovision release 4.8 software.

3D-SIM imaging was performed using a DeltaVision OMX 3D Structured Illumination Microscopy System® (Applied Precision Inc). Solid state lasers (405, 488, 593 nm) provided widefield illumination for simultaneous multichannel image capture using 3 Photometrics Cascade (Photometrics) back-illuminated EMCCD cameras (512×512 CCD, on-chip charge multiplication) and an Olympus UPlanSApo 100×1.4NA oil objective. 3D-SIM images were sectioned using a 125 nm Z-step size, and reconstructed as previously described [55], [56].

IEM of free, invading or post-invasion *P. falciparum* parasites were obtained via fixation of invasion preparations (as above) in 1% glutaraldehyde (ProSciTech, Australia) on ice for 30 min. Samples were pelleted in low-melt agarose (Bio-Rad Laboratories) before being transferred into water. After washing with water, samples were dehydrated with ethanol and embedded in LR Gold Resin (ProSciTech, Australia). Following polymerisation with benzoyl peroxide (SPI-Chem, USA) ultrathin sections (80–90 nm) were cut on a Leica Ultracut R ultramicrotome (Wetzlar). Immunolabeling was performed with mouse anti-HA (Roche Applied Science) clone 12AC5 [1∶100] or rabbit-anti HA (Sigma-Aldrich) [1∶10]. Samples were washed and incubated with 18 nm colloidal gold conjugated goat anti-mouse or anti-rabbit secondary antibodies (Jackson ImmunoResearch, Baltimore, USA) [1∶20]. Double labeling was carried out with a mixture of rabbit-anti HA (Sigma-Aldrich) [1∶10] and mouse anti-RAP1 [1∶200] primary antibodies. Samples were washed and incubated with secondary labels 18 nm and 12 nm colloidal gold conjugated goat anti-rabbit and anti-mouse antibodies (Jackson ImmunoResearch, Baltimore, USA) [1∶20]. After post-staining with 2% aqueous uranyl-acetate and 5% lead citrate sections were examined at 120 kV on a Philips CM120 BioTWIN Transmission Electron Microscope.

### General Image Processing

Reconstructed fluorescence deconvolution images and 3D-SIM images were rendered in 3D, with interpolation, using IMARIS version 7.2.2 (Bitplane Scientific). Whilst minimal adjustment of levels was used for presentation, no adjustment of gamma settings was undertaken. Images were assembled using Adobe Photoshop and Illustrator CS5 (Adobe Systems).

## Supporting Information

Text S1 **Supplemental materials and methods.**
(DOCX)

Figure S1 **PF14_0375 retains an asymmetrical localisation during merozoite invasion.** Widefield 3D imaging of PF14_0375-HA merozoites labeled with anti-HA, anti-PfRON4 and DAPI showing early invasion, mid and late invasion events. Scale bar = 5 µm. 3D reconstruction with 0.2 µm grid intervals.
(JPG)

Table S1 **Integrative genomic, transcriptomic and proteomic search strategy to indentify** ***P. falciparum*** **proteins conserved across** ***Plasmodium*** **spp. and** ***T. gondii*** **but absent in** ***Cryptosporidium*** **spp. and ookinete proteomic and EST data sets.**
(XLSX)

Table S2 **Ranking strategy of** ***P. falciparum*** **proteins from Table S1 to identify candidate invasins.**
(XLSX)
