# Supplementary material for: Subcompartmentalisation of Proteins in the Rhoptries Correlates with Ordered Events of Erythrocyte Invasion by the Blood Stage Malaria Parasite
Source: PLoS One. 2012 Sep 25;7(9):e46160. doi: 10.1371/journal.pone.0046160 (PMC3458004; doi:10.1371/journal.pone.0046160)
Supplement: Text S1 — Supplemental materials and methods. (DOCX) [file pone.0046160.s001.docx]

**Subcompartmentalisation of proteins in the rhoptries correlates with ordered events of erythrocyte invasion by the blood stage malaria parasite**

**Supplemental Experimental Procedures**

***Bioinformatic search strategy***

The data sources used for the integrative genomic, transcriptomic and proteomic invasin identification strategy are listed below with source availability (where online) and relevant publications.

| Data | Source | Publication |
| --- | --- | --- |
| *Plasmodium* spp. proteins | [www.PlasmoDB.org](http://www.PlasmoDB.org)  [www.geneDB.org](http://www.geneDB.org) | [1,2,3,4,5,6] |
| *Toxoplasma* *gondii* proteins | [www.ToxoDB.org](http://www.ToxoDB.org) | [7] |
| *Cryptosporidium* spp. proteins | [www.CryptoDB.org](http://www.CryptoDB.org) | [8,9,10] |
|  |  |  |
| Comparative orthologues | [www.OrthoMCL.org](http://www.OrthoMCL.org) | [11] |
| *P. berghei* ookinete proteome proteins |  | [12] |
| *P. gallinaceum* ookinete proteome |  | [13] |
| *P. berghei* ookinete micronemal proteome |  | [14] |
| *P. berghei* ookinete EST library |  | [15] |
| *Plasmodium* proteins | [www.PlasmoDB.org](http://www.PlasmoDB.org) | [5] |
| Maximum fold change of proteins through erythrocytic cycle (Affymetrix) | [www.PlasmoDB.org](http://www.PlasmoDB.org) | [5,16] |
| Maximum fold change of proteins through erythrocytic cycle (glass slide arrays) | [www.PlasmoDB.org](http://www.PlasmoDB.org) | [5,17] |
| Merozoite protein abundance | [www.PlasmoDB.org](http://www.PlasmoDB.org) | Leiden Malaria Group, unpublished data, [5] |
| Sporozoite protein abundance | [www.PlasmoDB.org](http://www.PlasmoDB.org) | [5,18] |
| Signal Peptide | SignalP | [19], |
| Transmembrane domain | TMHMM | [20] |
| PEXEL export motif | ExportPred | [21] |
| Malaria parasite protein localisation | [apiloc.bio21.unimelb.edu.au](http://apiloc.bio21.unimelb.edu.au) | Woodcroft B., et al, unpublished data |

***Immunofluorescence assay***

Primary antibodies used for IFA with *P. falciparum* and *P. berghei* parasites were rat anti-HA clone 3F10 (Roche Applied Bioscience) [1:100], rabbit anti-PfRON4 [1:250] [22], mouse anti-PfRON4 [1:500] [22], mouse anti-RAP1 [1:500] [23], rabbit anti-ACP [1:200] [24] (a kind gift from G. I. McFadden, University of Melbourne, Australia), rabbit anti-PfActin [1:400] [25], rabbit anti-MSP1-19 [1:250] [26] and rabbit anti-PfAMA1 [1:200] [27]. Primary antibodies with *T. gondii* parasites were rat anti-HA clone 3F10 [1:500] (Roche Applied Bioscience), mouse anti-GFP clones 7.1 and 13.1 [1:500] (Roche Applied Bioscience), rabbit anti-GFP [1:250] (Zymed Laboratories), rabbit anti-GAP45 [1:1000] [28], rabbit anti-SAG1 [1:1000] (a kind gift from L. D. Sibley), rabbit anti-RON4 [1:500] [29] and mouse anti-MIC2 [1:5000] (a kind gift from L. D. Sibley). Secondary antibodies [1:500] were Alexa Fluor® goat anti-rabbit 488 or 594, Alexa Fluor® goat anti-rat 488 or 594 and Alexa Fluor® goat anti-mouse 488 or 594.

**SUPPLEMENTAL REFERENCES**

1. Pain A, Bohme U, Berry AE, Mungall K, Finn RD, et al. (2008) The genome of the simian and human malaria parasite Plasmodium knowlesi. Nature 455: 799-803.

2. Carlton JM, Adams JH, Silva JC, Bidwell SL, Lorenzi H, et al. (2008) Comparative genomics of the neglected human malaria parasite Plasmodium vivax. Nature 455: 757-763.

3. Gardner MJ, Hall N, Fung E, White O, Berriman M, et al. (2002) Genome sequence of the human malaria parasite Plasmodium falciparum. Nature 419: 498-511.

4. Carlton JM, Angiuoli SV, Suh BB, Kooij TW, Pertea M, et al. (2002) Genome sequence and comparative analysis of the model rodent malaria parasite Plasmodium yoelii yoelii. Nature 419: 512-519.

5. Aurrecoechea C, Brestelli J, Brunk BP, Dommer J, Fischer S, et al. (2009) PlasmoDB: a functional genomic database for malaria parasites. Nucleic Acids Res 37: D539-543.

6. Logan-Klumpler FJ, De Silva N, Boehme U, Rogers MB, Velarde G, et al. (2012) GeneDB--an annotation database for pathogens. Nucleic Acids Res 40: D98-108.

7. Gajria B, Bahl A, Brestelli J, Dommer J, Fischer S, et al. (2008) ToxoDB: an integrated Toxoplasma gondii database resource. Nucleic Acids Res 36: D553-556.

8. Xu P, Widmer G, Wang Y, Ozaki LS, Alves JM, et al. (2004) The genome of Cryptosporidium hominis. Nature 431: 1107-1112.

9. Abrahamsen MS, Templeton TJ, Enomoto S, Abrahante JE, Zhu G, et al. (2004) Complete genome sequence of the apicomplexan, Cryptosporidium parvum. Science 304: 441-445.

10. Heiges M, Wang H, Robinson E, Aurrecoechea C, Gao X, et al. (2006) CryptoDB: a Cryptosporidium bioinformatics resource update. Nucleic Acids Res 34: D419-422.

11. Chen F, Mackey AJ, Stoeckert CJ, Jr., Roos DS (2006) OrthoMCL-DB: querying a comprehensive multi-species collection of ortholog groups. Nucleic Acids Res 34: D363-368.

12. Hall N, Karras M, Raine JD, Carlton JM, Kooij TW, et al. (2005) A comprehensive survey of the Plasmodium life cycle by genomic, transcriptomic, and proteomic analyses. Science 307: 82-86.

13. Patra KP, Johnson JR, Cantin GT, Yates JR, 3rd, Vinetz JM (2008) Proteomic analysis of zygote and ookinete stages of the avian malaria parasite Plasmodium gallinaceum delineates the homologous proteomes of the lethal human malaria parasite Plasmodium falciparum. Proteomics 8: 2492-2499.

14. Lal K, Prieto JH, Bromley E, Sanderson SJ, Yates JR, 3rd, et al. (2009) Characterisation of Plasmodium invasive organelles; an ookinete microneme proteome. Proteomics 9: 1142-1151.

15. Abraham EG, Islam S, Srinivasan P, Ghosh AK, Valenzuela JG, et al. (2004) Analysis of the Plasmodium and Anopheles transcriptional repertoire during ookinete development and midgut invasion. J Biol Chem 279: 5573-5580.

16. Le Roch KG, Zhou Y, Blair PL, Grainger M, Moch JK, et al. (2003) Discovery of gene function by expression profiling of the malaria parasite life cycle. Science 301: 1503-1508.

17. Bozdech Z, Llinas M, Pulliam BL, Wong ED, Zhu J, et al. (2003) The transcriptome of the intraerythrocytic developmental cycle of Plasmodium falciparum. PLoS Biol 1: E5.

18. Lasonder E, Janse CJ, van Gemert GJ, Mair GR, Vermunt AM, et al. (2008) Proteomic profiling of Plasmodium sporozoite maturation identifies new proteins essential for parasite development and infectivity. PLoS Pathog 4: e1000195.

19. Bendtsen JD, Nielsen H, von Heijne G, Brunak S (2004) Improved prediction of signal peptides: SignalP 3.0. J Mol Biol 340: 783-795.

20. Krogh A, Larsson B, von Heijne G, Sonnhammer EL (2001) Predicting transmembrane protein topology with a hidden Markov model: application to complete genomes. J Mol Biol 305: 567-580.

21. Sargeant TJ, Marti M, Caler E, Carlton JM, Simpson K, et al. (2006) Lineage-specific expansion of proteins exported to erythrocytes in malaria parasites. Genome Biol 7: R12.

22. Richard D, MacRaild CA, Riglar DT, Chan J-A, Foley M, et al. (2010) Interaction between Plasmodium falciparum apical membrane antigen 1 and the rhoptry neck protein complex defines a key step in the erythrocyte invasion process of malaria parasites. J Biol Chem 285: 14815-14822.

23. Schofield L, Bushell GR, Cooper JA, Saul AJ, Upcroft JA, et al. (1986) A rhoptry antigen of Plasmodium falciparum contains conserved and variable epitopes recognized by inhibitory monoclonal antibodies. Mol Biochem Parasitol 18: 183-195.

24. Waller RF, Keeling PJ, Donald RG, Striepen B, Handman E, et al. (1998) Nuclear-encoded proteins target to the plastid in Toxoplasma gondii and Plasmodium falciparum. Proc Natl Acad Sci U S A 95: 12352-12357.

25. Angrisano F, Riglar DT, Sturm A, Volz JC, Delves MJ, et al. (2012) Spatial localisation of actin filaments across developmental stages of the malaria parasite. PLoS One 7: e32188.

26. Boyle MJ, Wilson DW, Richards JS, Riglar DT, Tetteh KK, et al. (2010) Isolation of viable Plasmodium falciparum merozoites to define erythrocyte invasion events and advance vaccine and drug development. Proc Natl Acad Sci USA 107: 14378-14383.

27. Healer J, Crawford S, Ralph S, McFadden G, Cowman AF (2002) Independent translocation of two micronemal proteins in developing Plasmodium falciparum merozoites. Infect Immun 70: 5751-5758.

28. Gaskins E, Gilk S, DeVore N, Mann T, Ward G, et al. (2004) Identification of the membrane receptor of a class XIV myosin in Toxoplasma gondii. J Cell Biol 165: 383-393.

29. Alexander DL, Mital J, Ward GE, Bradley P, Boothroyd JC (2005) Identification of the Moving Junction Complex of Toxoplasma gondii: A Collaboration between Distinct Secretory Organelles. PLoS Pathog 1: e17.
