# Supplementary figures and images for: Subcompartmentalisation of Proteins in the Rhoptries Correlates with Ordered Events of Erythrocyte Invasion by the Blood Stage Malaria Parasite
Source: PLoS One. 2012 Sep 25;7(9):e46160. doi: 10.1371/journal.pone.0046160 (PMC3458004; doi:10.1371/journal.pone.0046160)

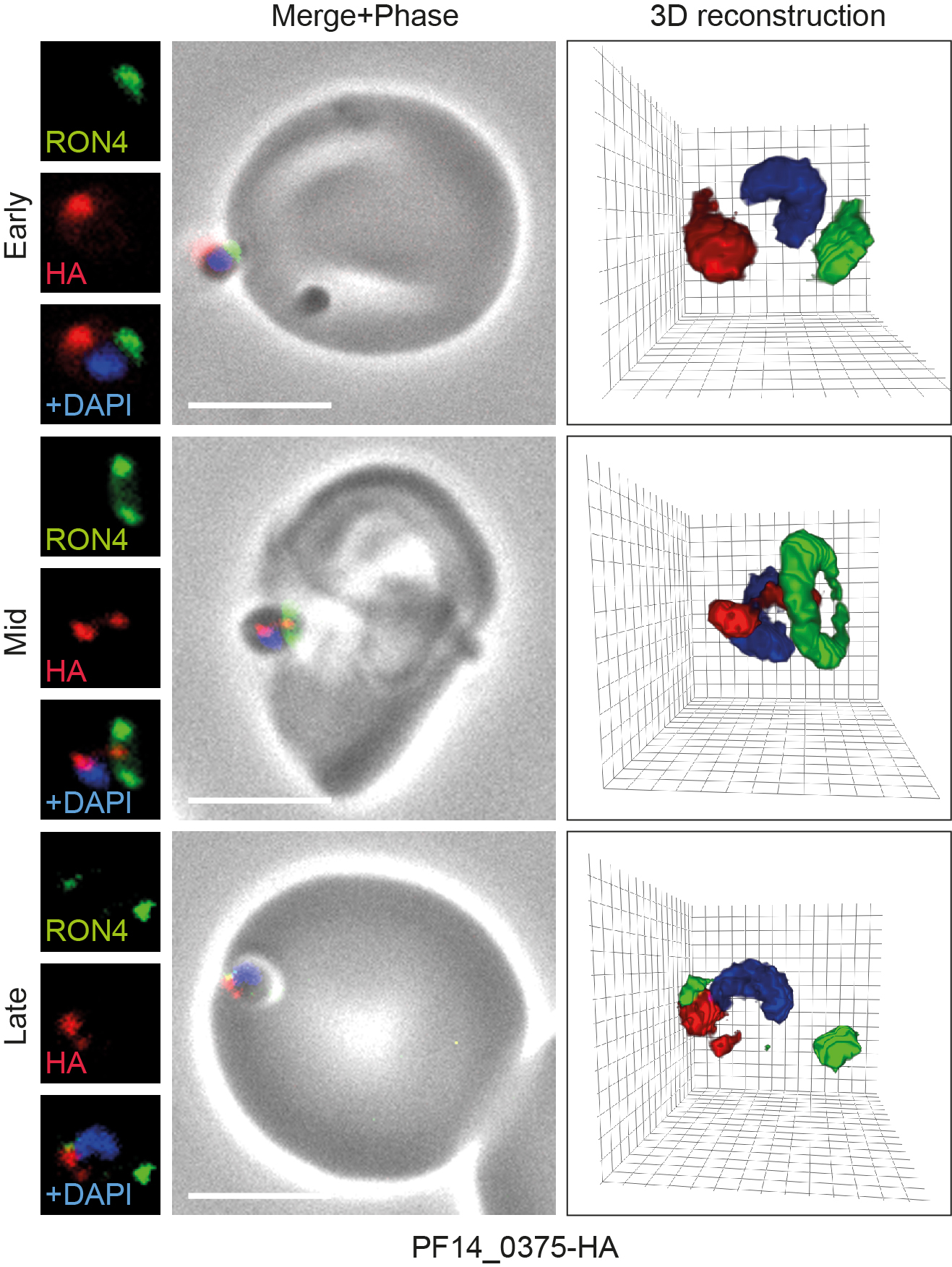

Supplement: Figure S1 — PF14_0375 retains an asymmetrical localisation during merozoite invasion. Widefield 3D imaging of PF14_0375-HA merozoites labeled with anti-HA, anti-PfRON4 and DAPI showing early invasion, mid and late invasion events. Scale bar = 5 µm. 3D reconstruction with 0.2 µm grid intervals. (JPG) [file pone.0046160.s002.jpg]
